# Chrysin-Induced Regression of Angiogenesis via an Induction of DNA Damage Response and Oxidative Stress in In Vitro and In Vivo Models of Melanoma

**DOI:** 10.3390/cells12121561

**Published:** 2023-06-06

**Authors:** Aicha Sassi, Maxime Fredon, Alexia K. Cotte, Camille Fuselier, Christophe Schneider, Laurent Martiny, David Monchaud, Leila Chekir-Ghedira, Virginie Aires, Dominique Delmas

**Affiliations:** 1 UFR Sciences de Santé, Université de Bourgogne, 21000 Dijon, France; aicha.sassi24@gmail.com (A.S.); maxime.fredon@efs.sante.fr (M.F.); alexiakm.cotte@gmail.com (A.K.C.); david.monchaud@u-bourgogne.fr (D.M.); virginie.aires02@u-bourgogne.fr (V.A.); 2 INSERM Research Center U1231—Cancer and Adaptive Immune Response Team, Bioactive Molecules and Health Research Group, 21000 Dijon, France; 3 Research Unit Bioactive Natural Products and Biotechnology UR17ES49, Faculty of Dental Medicine of Monastir, University of Monastir, Avicenne Street, Monastir 5000, Tunisia; leila.chekir@laposte.net; 4 Faculté des Sciences Exactes et Naturelles, UMR CNRS 7369 MEDyC, Université de Reims Champagne Ardenne, 51687 Reims, France; fuseliercamille@gmail.com (C.F.); christophe.schneider@univ-reims.fr (C.S.); laurent.martiny@univ-reims.fr (L.M.); 5 Institut de Chimie Moléculaire (ICMUB), CNRS UMR6302, UBFC, 21078 Dijon, France; 6 Centre de Lutte Contre le Cancer Georges François Leclerc Center, 21000 Dijon, France

**Keywords:** polyphenol, chrysin, cancer, melanoma, chemoprevention, DNA damage, angiogenesis

## Abstract

Despite the progress made in treatments, melanoma is one of the cancers for which its incidence and mortality have increased during recent decades. In the research of new therapeutic strategies, natural polyphenols such as chrysin could be good candidates owing to their capacities to modulate the different fundamental aspects of tumorigenesis and resistance mechanisms, such as oxidative stress and neoangiogenesis. In the present study, we sought to determine whether chrysin could exert antitumoral effects via the modulation of angiogenesis by acting on oxidative stress and associated DNA damage. For the first time, we show a link between chrysin-induced antiproliferative effects, the activation of the DNA damage pathway, and its ability to limit angiogenesis. More specifically, herein, we show that chrysin induces single- and double-stranded DNA breaks via the activation of the DNA damage response pathway: ATM (ataxia-telangiectasia-mutated)/Chk2 (checkpoint kinase 2) and ATR (ataxia telangiectasia and Rad3-related)/Chk1 (checkpoint kinase 1) pathways. Strong activation of this DNA damage response was found to be partly involved in the ability of chrysin to limit angiogenesis and may partly involve a direct interaction between the polyphenol and DNA G-quadruplex structures responsible for the replication fork collapse. Moreover, these events were associated with a marked reduction in melanoma cells’ capacity to secrete proangiogenic factor VEGF-A. The disruption of these key protein actors in tumor growth by chrysin was also confirmed in a syngeneic model of B16 melanoma. This last point is of importance to further consider the use of chrysin as a new therapeutic strategy in melanoma treatment.

## 1. Introduction

Despite the progress made in prevention, the early detection of at-risk populations, patient management, and treatment, the fight against cancer remains a major public health priority. In terms of mortality, cancer is responsible for one in eight deaths, and according to experts on a global scale, its incidence should reach 21.4 million cases per year by 2030. These therapeutic failures are notably linked to the emergence of intrinsic or acquired resistance by the tumors. Research in recent years has identified some molecular mechanisms that contribute to resistance [1]. Among these mechanisms, neoangiogenesis, as well as genomic instability associated with alterations in DNA damage response pathways underlying unrestricted tumor cell proliferation, seems to be substantially linked. Despite these advances, recently developed targeted therapies show only moderate objective responses on overall patient survival due to the development of resistance. Therefore, there is still a need to develop new therapeutic strategies to prevent the development of cancers, increase the sensitivity of tumors relative to existing therapies, or identify new therapeutic targets to counteract resistance phenomena.

In this strategy of prevention and chemosensitization, natural molecules such as polyphenols appear to be good candidates due to their low toxicity and specificity toward tumor cells. Indeed, numerous in vitro and in vivo studies have shown that these natural molecules are able to block all stages of carcinogenesis and sensitize tumor cells relative to many anticancer agents, thus increasing their chemotherapeutic potential [2,3]. At the cellular and molecular level, polyphenols can act at the DNA level and modulate many signaling pathways involved in cell death, inflammation, angiogenesis, or even in metabolism [4,5,6,7,8]. Moreover, polyphenols, due to their capacity to act as anti- and pro-oxidant molecules [5], could also contribute to limiting some fundamental aspects of tumorigenesis and resistance mechanisms by acting on the cellular redox balance. Indeed, oxidative stress and reactive oxygen species (ROS) have been widely described as contributing to cancer initiation and development, especially in melanoma [9,10,11]. In the early stages of oncogenesis, they contribute to genomic instability and malignant transformation by altering DNA repair pathways. In later stages, due to altered tumor metabolism and pro-inflammatory microenvironment, they contribute to cell proliferation, migration, invasion, angiogenesis, and metastasis dissemination [12].

**Figure 1:**
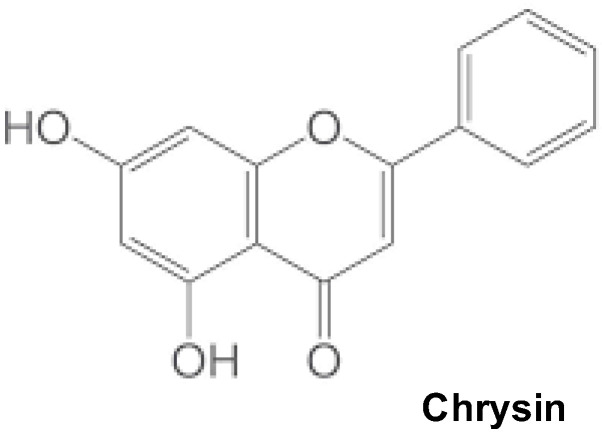
Chemical structure of chrysin (5,7-dihydroxyflavone).

Among polyphenols that could be possible anticarcinogenic agents, recent studies have highlighted the potential of a 5,7-dihydroflavone flavanone, chrysin (Figure 1).

This natural flavonoid extracted from honey, propolis, or blue passionflower could be an interesting candidate due to its chemopreventive and therapeutic properties [6]. Indeed, many studies tend to show that chrysin can target the carcinogenesis process by modulating multiple signaling pathways involved in inflammation, proliferation, death and tumor invasion [13,14]. In addition, chrysin could be used in chemosensitization strategies as a therapeutic adjuvant following recent work showing its ability to potentiate the action of anticancer agents such as doxorubicin, cisplatin, or ciglitazone [15], and it could be used to reduce tumor angiogenesis in a nude mouse model xenografted with prostate tumor cells [16], as previously shown for many other natural compounds [8,17,18]. Then, such a compound could help in addition to conventional chemotherapy against certain cancers that exhibit very low survival prognoses and for which resistance phenomena often emerge. Among the cancers for which their incidence and mortality have been steadily increasing in recent years, melanoma is one of the cancers with a very poor vital prognosis in advanced stages, with only 15% survival at 5 years regardless of the treatment. Moreover, metastatic melanoma is a type of cancer that is not very sensitive to conventional therapies and targeted therapies due to the establishment of numerous resistance mechanisms, including very significant vascularization and a defect in the induction of the response to damage to the DNA. Additionally, due to having properties similar to other polyphenols such as resveratrol, chrysin could limit tumor angiogenesis by acting on the response to DNA damage via the modulation of oxidative stress.

In the present study, we hypothesized that chrysin was able to induce DNA damage by targeting single- and double-stranded DNA via activation of the DNA damage response (DDR) pathway: ATM (ataxia-telangiectasia-mutated)/Chk2 (checkpoint kinase 2) and ATR (ataxia telangiectasia and Rad3-related)/Chk1 (checkpoint kinase 1) pathways. This chrysin action could involve binding to DNA structures via interaction with G quadruplex (G4) and duplex structures. Furthermore, by using an in vitro model of endothelial cells such as human umbilical vein endothelial cells (HUVECs) and an in vivo syngeneic model of B16 melanoma, we investigated whether the ability of chrysin to act on the DNA damage pathway is correlated with its ability to decrease angiogenesis. This last point is very important for the consideration of the potential antitumoral effect of chrysin when entering new therapeutic strategies against melanoma.

## 2. Materials and Methods

### 2.1. Cell Lines

Three human and murine melanic lines from the “American Type Culture Collection” (ATCC) were used: the SK-ML-28 line established from a primary skin tissue tumor from a Caucasian human, the MelC line derived from a grade IV human metastatic melanoma tumor, and the murine B16F10 line derived from lung metastases in mice (C57BL/6jRJ). The SK-ML-28 and MelC cell lines have an activating mutation of the BRAF serine/threonine kinase protein (BRAFV600E), while the B16F10 cells express the wild-type form of BRAF. These three cell lines do not possess mutations of the p53 protein and express the wild-type form of the protein. The cultured cells were maintained in Dulbecco’s Modified Eagle F12 medium (DMEM/F12) supplemented with 10% fetal bovine serum (Dutscher, Brumath, France) and 1% penicillin/streptomycin in a humidified atmosphere of 5% CO_2_ at 37 °C.

### 2.2. Drugs and Chemical Reagents

The chrysin stock solution (Sigma Aldrich^®^, # C80105, St. Louis, MO, USA) was prepared at 100 mM in DMSO and then diluted in the culture medium at the indicated concentrations containing a final quantity of 0.1% DMSO. ATM (ATMi, KU55933, Sigma Aldrich^®^) and ATR (ATRi, KU55933, Tocris^®^, Bristol, UK) protein inhibitors were used at a final concentration of 10 μM. Reduced L-glutathione, GSH (G2451, Sigma Aldrich^®^), is used at 10 mM in the final concentration, and hydrogen peroxide (H_2_O_2_) was used at final concentrations of 20 and 40 µM.

### 2.3. Viability Assays

The viability assays were assessed by crystal violet staining (Sigma Aldrich, St. Quentin Fallavier, France). Briefly, SK-ML-28 (3400 cells/well), MelC (6000 cells/well), and B16F10 (3400 cells/well) cells were seeded 24h before treatment in 96-well plates in a complete growth medium. The next day, cells were treated with increasing concentrations of chrysin (0 to 200 µM) for 72 h. All control and treated cells received the same amount of DMSO (0.1%). At the end of treatment, cells were washed with phosphate-buffered saline (PBS), fixed with ethanol for 10 min at 4 °C, and then stained with a crystal violet solution (0.5% (*w*/*v*) crystal violet in 25% (*v*/*v*) methanol) for 15 min at room temperature. Cells were then gently rinsed with water, and absorbance was measured at 590 nm using a Biochrom Assay UVM 340 microplate reader following the extraction of the dye using a 33% acetic acid solution.

### 2.4. Apoptosis Detection

For apoptosis assays, cells were seeded in 6-well plates at a density of 1 × 10^5^ for SK-ML-28 and B16F10 and 4 × 10^5^ for MelC cells. Apoptosis was determined using annexin V-FITC and 7-aminoactinomycin D (7AAD) staining from BD Biosciences according to the manufacturer’s instructions. The data were analyzed using FlowJo^®^ software (Tristar, v10.0.7), and the percentages of cells in early apoptosis (Annexin V+/7-AAD− cells) and late/or necrosis (Annexin V+/7-AAD+ cells) were quantified as previously described [19].

### 2.5. Western Blot Analysis

Melanic cells were seeded in 75 cm^2^ flasks at a density of 1 ×10^6^/flask for SK-ML-28 and B16F10 and 2 × 10^6^ cells/flask for MelC cells. After treatments with chrysin (0, 20, 40, and 80 µM) for 72 h, cells were either washed with cold 1X phosphate-buffered saline (PBS Dutscher, Brumath, France) or lysed in a boiling buffer (SDS sodium dodecyl sulfate 1%, orthovanadate 1 mM, Tris 10 mM (pH 7.4)) with protease inhibitors (Roche Applied Bioscience) for 30 min on ice. Then, the lysates were sonicated for 6/7 s at 30% amplitude. Protein concentrations were measured using a BCA assay kit (Thermo Fisher Scientific, Waltham, MA, USA). In this study, 25 to 60 μg of total protein was loaded onto 10% polyacrylamide gel. Proteins were resolved by SDS-PAGE and transferred to nitrocellulose membranes (Amersham). Blots were then saturated in 5% milk (1 h at RT) before overnight incubation at 4 °C with specific primary antibodies (Supplementary Table S1). All primary antibodies were diluted at 1:1000 in 5% *w*/*v* non-fat milk or 5% BSA. Primary antibodies were detected using appropriate horseradish peroxidase (HRP)-conjugated secondary antibodies (Cell Signaling Technologies, Danvers, MA, USA) followed by exposure to ECL (Santa Cruz Biotechnology, Dallas, TX, USA). The signal was acquired using a ChemiDoc^TM^ XRS+ imaging system (Biorad, Marnes-la-Coquette, France), and blots were analyzed using Image Lab^TM^ Software 5.1.2 (Biorad, Marnes-la-Coquette, France).

### 2.6. Quantification of DNA Damage by Flow Cytometry

For flow cytometry experiments, cells were seeded in 6-well plates at a density of 1 × 10^5^ for SK-ML-28 and B16F10 and 4 × 10^5^ for MelC cells. The quantification of DNA damage was performed by flow cytometry after labeling phosphorylated histone H2AX on Serine 139 (pS139-H2AX or γ-H2AX). After treatment, the cells are fixed in 100% icy methanol for 15 min, washed in 1X PBS, and then permeabilized and saturated for 20 min with a 1X PBS/BSA (Bovine serum albumin) 1%/Triton-X100 solution. After incubation for 1 h with a primary rabbit antibody directed against the pS139-H2AX protein (diluted to 1/1000th in 1X PBS, 1% BSA; Santa Cruz biotechnology, Sc-101696), the cells are washed with PBS 1X and then incubated for 1 h with a secondary antibody coupled to the Alexa488 fluorochrome (diluted to 1/500th in a solution of 1X PBS, 1% BSA, Invitrogen/Molecular Probes^®^, Eugene, OR, USA). Finally, the cells are washed and incubated for 30 min with a solution of propidium iodide (PI: 50 μg/mL) in the presence of 200 μg/mL of RNAse A before being analyzed using flow cytometry (BD FACS Canto II Flow Cytometer, BD Biosciences^TM^, Franklin Lakes, NJ, USA) with the following parameters: PI 488 nm excitation and 576/26 emission filter and Alexa 488 (γH2AX staining) 488 nm excitation and 530/30 emission filter. With an overlap of the two emission spectra, compensations were provided using mono-labeled cells. In both cases, non-specific labeling was evaluated using a non-relevant antibody (IgG alone). Cytometry data analysis was performed using FlowJo^®^ software (v10.0.7).

### 2.7. Immunofluorescence

Melanic cells were seeded 24 h before treatment in 24-well plates on sterile glass slides at a density of 2 × 10^4^ cells/well for SK-ML-28 and B16F10 cells and at a density of 4 × 10^4^ cells/well for MelC. The next day, the cells were treated or not with chrysin. After 72 h of treatment, the cells were washed twice with 1X PBS, fixed for 15 min in icy methanol at 4 °C, and then rehydrated by washing with 1X PBS. Cells were then permeabilized and saturated for 20 min at room temperature with a solution of 1X PBS/0.2% Triton-X100/1% BSA. After 1.5 h of incubation at room temperature with the primary antibody directed against γH2AX (diluted to 1/500th in 1X PBS, 0.2% Triton X-100, 1% BSA; Santa Cruz, Sc-101696), the cells were washed and then incubated for 1.5 h in the dark with the secondary Alexa488 antibody (Invitrogen/Molecular Probes; λ excitation/λ emission: 495/520 nm) diluted to 1/500th in PBS 1X/Triton-X100 0, 2%/BSA 1%. After washing, the coverslips containing the cells were transferred to glass slides containing ProLong^®^ Gold Antifade mounting medium (Invitrogen/Molecular Probes) and counterstained with DAPI (4′,6′-Diamidino-2-Phenylindole; λ excitation/λ emission: 358/461 nm). The slides were then dried overnight at 4 °C before analysis by fluorescence microscopy (Axio Imager M2 Zeiss, Jena, Germany, ×40 objective) using Zen Blue^TM^ software (v2.3).

### 2.8. Interaction Measurement with DNA G-Quadruplex/Duplex

Cell-free in vitro assays were performed based on the displacement of a fluorescent DNA probe, thiazole orange (TO). TO is highly fluorescent when complexed with DNA and is non-fluorescent when free in a solution, making TO an interesting molecule for labeling different DNA structures (single strands, duplexes, and quadruplexes) and for the rapid evaluation of potential ligands to DNA [20]. If a compound is likely to interact with DNA, it competes with TO and displaces it, resulting in a decrease in fluorescence markings as the compound has a high affinity for DNA. DNA quadruplexes (G4) and duplexes (ds26) were prepared in a CacoK buffer (10 mM lithium cacodylate buffer (pH 7.2), 10 mM KCl, 90 mM LiCl) according to [20]. The DNA sequences used for this study were obtained from Eurogentec and are listed as follows: G4, d [5′AGGGTTAGGGTTAGGGTTAGGG3′] and ds26, d[5′CAATCGGATCGAATTCGATCCGATTG3′]. The assays were performed by mixing the DNA templates (G4 or ds26, 0.25 µM in a final volume of 1 mL CacoK buffer) with 1 µM of TO (Sigma Aldrich, #390062), and then the ligands were added in equivalent DNA amounts. From this mixture, two tests were performed: (1) measurement of TO fluorescence quenching by the addition of increasing concentrations of ligands after a 3 min equilibration period and (2) measurement over time of TO fluorescence quenching by a fixed concentration of ligands. In both cases, the TO fluorescence spectrum was evaluated by spectrofluorimetry (excitation: 510 nm; emission: 510–750 nm). The percentage shift of TO is calculated from the fluorescence spectrum (FA) using the following formula: % shift = 100 − [(FA/FA0) × 100], where FA0 is the fluorescence of TO bound to DNA without the addition of ligand. The tested ligands were chrysin and quercetin, which is a polyphenol described to be a ligand for DNA quadruplexes [21], and it was used as a positive control.

### 2.9. Oxidative Stress Assays

For oxidative stress experiments, cells were seeded in 6-well plates at a density of 1 × 10^5^ for SK-ML-28 and B16F10 and 4 × 10^5^ for MelC cells. To measure the production of ROS, we used probes comprising 2′,7′-dichlorodihydrofluorescein diacetate (H_2_DCFDA) (Invitrogen/Molecular Probes^®^), which fluoresces when oxidized by intracellular ROS and MitoSOX (MitoSOX™, M36008, Molecular Probes^®^), which then responds to mitochondrial O_2_^−^ (both obtained from Molecular Probes). Briefly, cells were incubated with 10 µM H_2_DCFDA or MitoSOX for 15 min, washed with PBS, and immediately analyzed by using flow cytometry. All assays were analyzed using flow cytometry on a BD LSR-II cytometer equipped with BD FACSDiva software (BD Biosciences).

### 2.10. RNA Extraction and Quantitative PCR Analysis

Total cellular RNA was extracted using TRIzol^®^ RNA Isolation Reagent (Ambion, Austin, TX, USA). RNA (300 ng) was reverse-transcribed into cDNA using M-MLV reverse transcriptase, random primers, and an RNAseOUT inhibitor (Invitrogen, Waltham, MA, USA). cDNA was quantified by real-time PCR with the Power SYBR Green PCR Master mix (Applied Biosystems; Warrington, UK) on a 7500 Fast Real-Time PCR detection system (Applied Biosystems). Relative mRNA levels were determined by the ΔΔCt method and normalized to the expression levels of human or mouse Actb (the primer sequences used are listed in Supplementary Table S2).

### 2.11. ELISA

For these experiments, cells were seeded in 6-well plates at a density of 1 × 10^5^ for SK-ML-28 and B16F10 and 4 × 10^5^ for MelC cells. Cell culture supernatants were assayed by ELISA for human VEGF-A (BMS277-2 Invitrogen), according to the manufacturer’s protocol.

### 2.12. Tumor Growth Analyses In Vivo

Ten 8-week-old female C57BL/6JRj mice (Janvier Labs) were divided into two groups: a control group (five mice) and a treated group (five mice). All mice were housed and maintained in a designated pathogen-free area accredited by the Federation of Laboratory Animal Science Associations (FELASA) in accordance with the University of Champagne-ardenne’s Animal Experimental Ethics Committee guidelines (CEEA-RCA n°56). The mice received a standard diet ad libitum, and it was supplemented or not daily with chrysin at a rate of 50 mg/kg. Animals were individually treated per os by administrating an appropriate amount of chrysin. This supplementation was carried out 15 days (D-15) before the subcutaneous inoculation of 200,000 B16F10 cells on the right flank of the mice, and it was maintained for 16 days until the animals were euthanized. Tumor volumes were then determined on D9, D11, D14, and D16 by using a caliper. After 14 days of tumor growth, a contrast agent (Viscover Exitron nano 12,000 CT, Miltenyi Biotec, Bergisch Gladbach, Germany) was injected retro-orbitally into mice anesthetized under isoflurane in order to visualize tumor vascularization using a micro-scanner X-ray (SkyScan, Bruker, Billerica, MA, USA). Then, mice were sacrificed on D16 to remove the tumors. These were cut into 2 homogeneous parts in order to analyze the presence and quantity of different key players that have a role in DDR and angiogenesis by immunoblotting and using RT-qPCR. In order to assess the impact of chrysin supplementation on angiogenesis via the measurement of VEGF-A secretion, part of the tumors was dissociated with gentle MACSTM Dissociator (Miltenyi Biotec) using a specific dissociation of murine tumors (Tumor Dissociation kit mouse, Miltenyi Biotec, 130-096-730) according to the supplier’s recommendations. After dissociation, the supernatant was recovered for the VEGF-A assays by ELISA.

### 2.13. Densitometry and Statistical Significance

The densitometry of blots was realized by the use of ImageJ software (National Institutes of Health). Statistical analyses were only carried out in experiments that were independently performed three times, each including a minimum of three replicates per experimental condition. These analyses were performed using the statistical module of Prism software (GraphPad Software, v6.07). For the analysis of experimental data, continuous variables were compared using the Mann–Whitney U test, multiple Student’s *t*-tests, and univariate or bivariate ANOVA tests, followed by Bonferroni’s or Tukey’s post hoc tests after checking the normal distribution of the data and the homogeneity of variances. All probability (*p*) values are two-sided. *p* values less than 0.05 were considered significant (* *p* < 0.05; ** *p* < 0.01; *** *p* < 0.001; **** *p* < 0.0001). Data presented are means ± standard deviation (SD) for in vitro experiments, and they are presented as mean ± standard error of the mean (SEM) for in vivo experiments.

## 3. Results

### 3.1. Chrysin Prevents Melanoma Cell Proliferation

**Figure 2:**
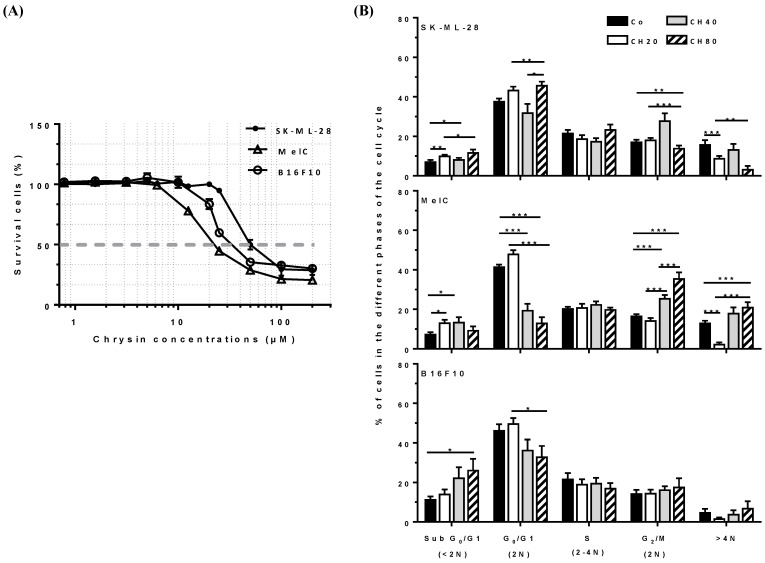
Chrysin affects melanic cell proliferation via a disruption of the cell cycle’s progression. (**A**) Melanic cells, SK-ML-28, MelC, and B16F10, were treated with a medium containing the solvent (0.1% DMSO) or a game range of chrysin (0 to 200 µM) at 37 °C for 72 h; the percentage of cell viability was determined by a crystal violet assay. Results are expressed as a percentage of the control. Data are the mean ± S.D. of four independent experiments with *n* = 10. The grey dot line indicates the chrysin IC_50_. (**B**) Quantitative analysis of cell cycle distribution in melanic cells treated with medium containing the solvent (0.1% DMSO) or with chrysin. Data are expressed as mean ± SEM of three independent experiments; *p* values were determined by a two-way ANOVA followed by Dunnett’s multiple comparison test. * *p* < 0.05; ** *p* < 0.01; and *** *p* < 0.001 compared to control (Co) conditions.

We and others have previously shown that chrysin was able to inhibit the proliferation of B16F10 murine melanoma cells [22,23]. In order to observe if chrysin exerts an action regardless of the mutation status of the cancer line, we used several melanoma cell lines with different statuses such as MelC (grade IV metastatic human melanoma), SK-ML-28 cells (primary tumor), and B16F10 (metastatic murine melanoma). We first determined the cytotoxic effect of chrysin by using crystal violet, and we observed that this flavonol induced a more cytotoxic effect against MelC and B16F10 than SK-ML-28 (Figure 2A). Indeed, the 50 percent inhibitory concentration of tumor cells (IC_50_) was 25 ± 5 µM for MelC and 27 ± 2.6 µM for B16F10 cells compared with 46.67 ± 5.8 µM for SK-ML-28. Therefore, chrysin is active on the three tumor cell lines, with greater sensitivity to MelC and B16F10 cells compared to SK-ML-28; this is why we used three concentrations for the following experiments (20, 40, and 80 µM of chrysin), framing the obtained IC_50_ with the three cell lines.

### 3.2. Chrysin Disrupts Cell Cycle Progression at the G_2_/M Phase and Induces Tetraploid Cells

As previously shown with many polyphenols, such as resveratrol, their cytotoxic effects were often associated with a disruption of the cycle at different phases [8,18,24,25]. Therefore, to assess whether chrysin-induced antiproliferative effects could be associated with a disruption of cell cycle progression, melanoma cells were exposed for 72 h with a game range of chrysin (20, 40, and 80 µM) before the addition of BrdU in the last hour of treatment. Cells with high fluorescence were S-phase cells that incorporated BrdU. Those with background fluorescence represented G_1_ or G_2_/M phase cells depending on DNA content (2C or 4C, respectively). The results reveal that chrysin mainly induces effects during an arrest in the G_2_/M phase in the cell lines (Figure 2B). Indeed, the quantification of cell distribution shows that chrysin at 40 µM induces a strong increase in SK-ML-28 and MelC in the G_2_/M phase starting from +61% and +53%, respectively. This effect in the MelC cell line is accentuated with a high concentration of chrysin (80 µM), which induces an accumulation of +112% (Figure 2B). Moreover, this progression disruption of the cell cycle induced by chrysin is also associated with a decrease in melanoma MelC cell numbers in the G_0_/G_1_ phase of −53% and −68% relative to 40 and 80 µM of flavonol in a concentration-dependent manner (Figure 2B), respectively. These tendencies to accumulate cells in the G_2_/M phase and significantly decrease cells in the G_0_/G_1_ phase were also observed in B16F10 cells.

Very interestingly, chrysin led to the appearance of an important population of cells with a DNA content higher than 4N, indicating the presence of polyploid cells in the tested cell lines (Figure 2B). Indeed, we observed an increase of +37% and +60% with 40 and 80 µM in MelC cells and an increase of +40% with 80 µM of chrysin in B16F10 cells, respectively. This polyploidy can result from a mechanism of DNA endoreplication without cytokinesis. Indeed, a cell cycle arrest might be due to the induction of DNA lesions or damage, which are detected by a signaling cascade called DDR and normally leads to DNA repair. If the repair mechanisms are ineffective or if the intensity of the damage is too high, the cells evolve toward death, notably by apoptosis.

### 3.3. Chrysin Induces DNA Damages, Leading to the Apoptosis Process

**Figure 3:**
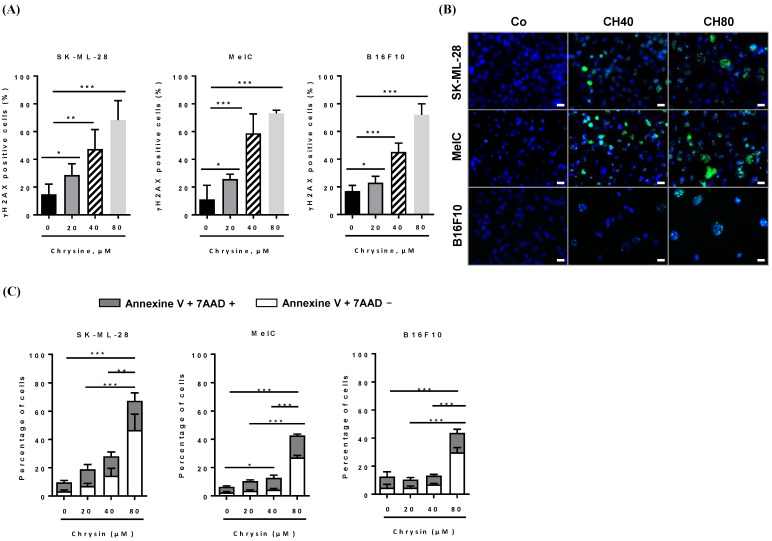
Chrysin induces the overexpression of phosphorylated histone H2AX, reflecting DNA damage. (**A**) Quantification of the percentage of γH2AX-positive cells of 3 independent experiments (*n* = 9). Significant differences between treatments were determined by ANOVA followed by Tukey’s post hoc test. * *p* < 0.05; ** *p* < 0.01; *** *p* < 0.001. (**B**) Tumor cells were treated with a medium containing the solvent (0.1% DMSO) or with chrysin (20, 40, and 80 µM) for 72 h before the fluorescent immunostaining of the γH2AX protein (green staining) and against the staining of cell nuclei with Hoechst 33,342 (blue staining). Images are representative of 3 independent experiments (*n* = 3, ×40 objective). White bars correspond to 10 µm. (**C**) Melanic cells were treated in a medium containing the solvent (0.1% DMSO) or with 20, 40, and 80 µM chrysin for 72 h, and then the cells were labeled with Annexin V/7AAD to assess the percentage of cells in early (Annexin V+/7AAD− cells) and late (secondary necrosis, Annexin V+/7AAD+ cells) apoptosis. Histograms represent mean percentages ± SD corresponding to three independent experiments per cell line, with three replicates per concentration each time. The *p* values were determined by a two-way ANOVA test with Bonferroni correction. * *p* < 0.05; ** *p* < 0.01; *** *p* < 0.001.

We have previously demonstrated that polyploidy with another polyphenol (i.e., resveratrol) in colon cancer cell lines led to both apoptosis and senescence [8,24]. To determine whether the chrysin-induced accumulation of tumor cells in the G_2_/M phase can be associated with an induction of DNA damage and cell death, we first performed double labeling on γH2AX/IP and quantified the percentage of cells that are positive for this marker by using flow cytometry. The phosphorylated H2AX histone, γH2AX, is usually used to assess DNA damage levels, and it is a marker of single- and double-stranded DNA breaks [26,27]. After 72 h of chrysin treatment, our results revealed that most diploid and tetraploid melanoma cells are γH2AX-positive in the three cell lines (Figure 3A). The quantification revealed that chrysin induced a strong expression of γH2AX starting at 40 µM where SK-ML-28 exhibited high levels of +50 and +70% at 40 and 80 µM and +60% and +70% in MelC. Similar results were obtained in B16F10 with increases of +28% and +55% relative to 40 and 80 µM of chrysin, respectively (Figure 3A).

Microscopic analyses highlighted typical γH2AX foci after chrysin treatments, which are associated with positive apoptotic cell counterstaining with Hoechst 33,342 (i.e., condensation and fragmentation of the nuclear chromatin) (Figure 3B). These microscopic results are in accordance with increases in the percentage of melanic cells observed previously by a flow cytometer in the subdiploid phase (Sub G_0_/G_1_) induced by chrysin (Figure 2B). To support the induction of cell apoptosis by chrysin, we performed a more accurate quantification using double staining with Annexin V/7-amino-actinomycin D (7-AAD), which allows for the discrimination between early apoptotic cells (Annexin V+/7-AAD−) and late apoptotic cells (Annexin V+/7-AAD+), since cells can no longer exclude 7-AAD in advanced stages, which then bind to DNA with a high affinity. Flow cytometry analysis then showed that chrysin significantly induced cell death with a high level of early apoptosis, especially after 72 h of treatment with 80 µM, in all three lines (Figure 3C).

### 3.4. Chrysin Induces DNA Damage in Melanic Cell Lines

**Figure 4:**
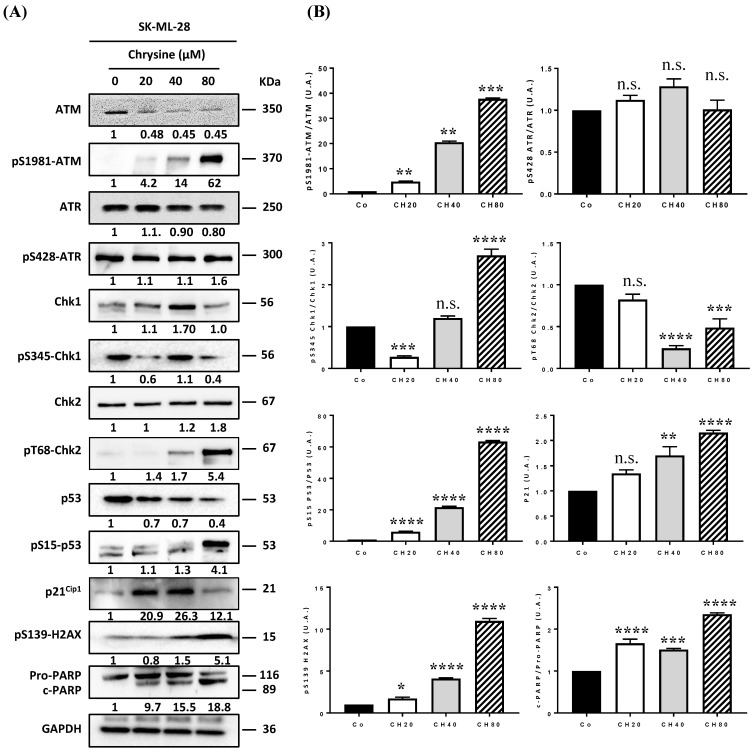
(**A**) Chrysin disrupts the ATM/ATR signaling pathway. (**A**) Immunoblot analysis of ATM, phospho-ATM (pS1981-ATM), ATR, phosphor-ATR (pS428-ATR), Chk1, phospho-Chk1 (pS345-Chk1), Chk2, phosphor-Chk2 (pT68-Chk2), p53, phosphor-p53 (pS15-P53), p21^Cip1^, phosphor-p21^Cip1^, phosphor-H2AX (pS139-H2AX), Pro-PARP, and cleaved PARP (c-PARP) in SK-ML-28 cells treated with a medium containing the solvent (0.1% DMSO) or with increasing concentrations (20, 40, and 80 µM) of chrysin for 72 h. GAPDH was used as a loading control. The numbers below indicate the average value of the intensity of the bands of 3 independent immunoblotting operations compared to the solvent control set at a value of 1. (**B**) Densitometry quantification of Western blotting. Data are expressed as the mean fold induction ± SEM of three independent experiments. *p* values were determined by one-way ANOVA followed by Tukey’s multiple comparison test. * *p* < 0.05; ** *p* < 0.01; *** *p* < 0.001; and **** *p* < 0.0001; n.s.: not significant.

**Figure 5:**
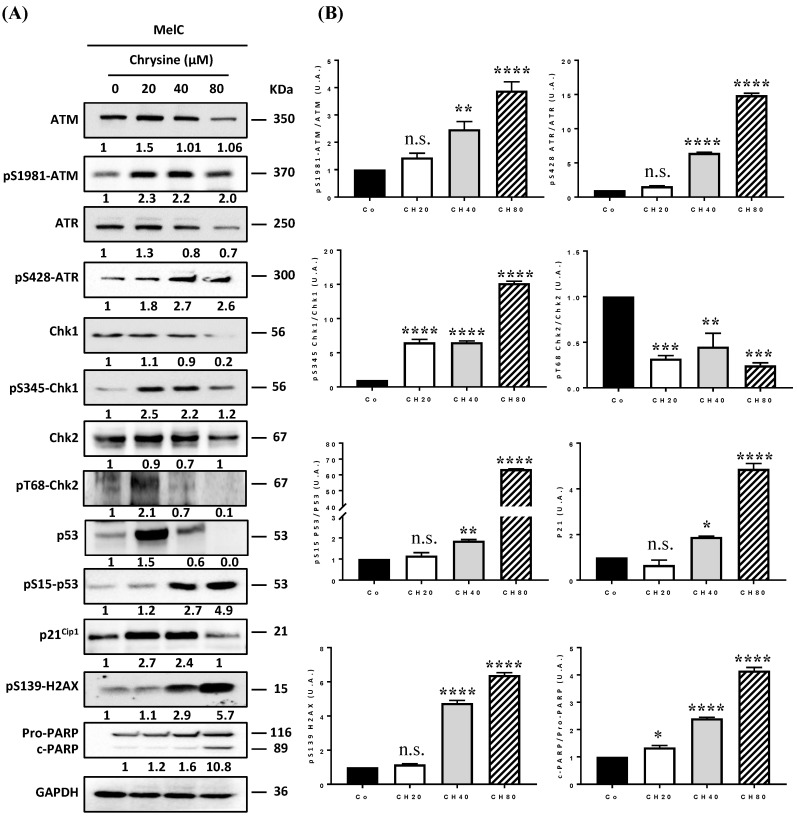
Chrysin disrupts the ATM/ATR signaling pathway. (**A**) Immunoblot analysis of ATM, phospho-ATM (pS1981-ATM), ATR, phosphor-ATR (pS428-ATR), Chk1, phospho-Chk1 (pS345-Chk1), Chk2, phosphor-Chk2 (pT68-Chk2), p53, phosphor-p53 (pS15-P53), p21^Cip1^, phosphor-p21^Cip1^, phosphor-H2AX (pS139-H2AX), Pro-PARP, and cleaved PARP (c-PARP) in MelC cells treated with a medium containing the solvent (0.1% DMSO) or with increasing concentrations (20, 40, and 80 µM) of chrysin (CH) for 72 h. GAPDH was used as a loading control. The numbers below indicate the average value of the intensity of the bands of 3 independent immunoblotting operations compared to the solvent control set at a value of 1. (**B**) Densitometry quantification of Western blotting. Data are expressed as the mean fold induction ± SEM of three independent experiments. *p* values were determined by one-way ANOVA followed by Tukey’s multiple comparison test. * *p* < 0.05; ** *p* < 0.01; *** *p* < 0.001; and **** *p* < 0.0001; n.s.: not significant.

**Figure 6:**
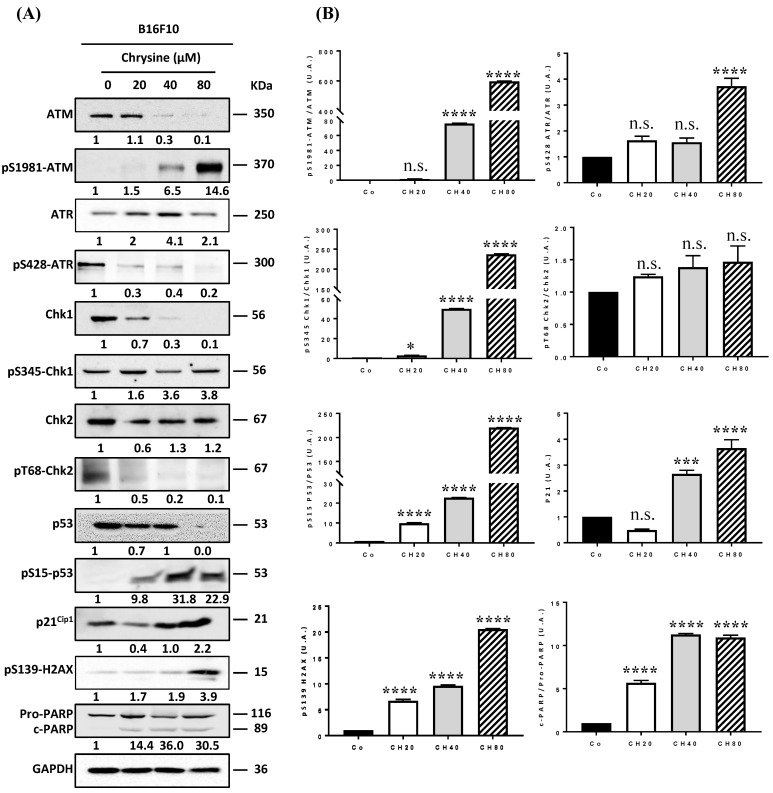
Chrysin disrupts the ATM/ATR signaling pathway. (**A**) Immunoblot analysis of ATM, phospho-ATM (pS1981-ATM), ATR, phosphor-ATR (pS428-ATR), Chk1, phospho-Chk1 (pS345-Chk1), Chk2, phosphor-Chk2 (pT68-Chk2), p53, phosphor-p53 (pS15-P53), p21^Cip1^, phosphor-p21^Cip1^, phosphor-H2AX (pS139-H2AX), Pro-PARP, and cleaved PARP (c-PARP) in B16F10 cells treated with a medium containing the solvent (0.1% DMSO) or with increasing concentrations (20, 40, and 80 µM) of chrysin for 72 h. GAPDH was used as a loading control. The numbers below indicate the average value of the intensity of the bands of 3 independent immunoblotting operations compared to the solvent control set at a value of 1. (**B**) Densitometry quantification of Western blotting. Data are expressed as the mean fold induction ± SEM of three independent experiments. *p* values were determined by one-way ANOVA followed by Tukey’s multiple comparison test. * *p* < 0.05; *** *p* < 0.001; and **** *p* < 0.0001; n.s.: not significant.

DNA damage, whether single- or double-stranded breaks, results in the activation of cell cycle checkpoints, leading to cell cycle arrest and the activation of the DDR pathway. The two main pathways activated as a result of genomic damage are the ATM (ataxia-telangiectasia-mutated)/Chk2 (Checkpoint kinase 2) and ATR (ataxia telangiectasia and Rad3-related)/Chk1 (checkpoint kinase 1) pathways. ATM and ATR proteins phosphorylate downstream histone H2AX (γH2AX), which then recruits other ATM and ATR substrates, notably Chk2 and Chk1 proteins. These proteins can then modulate the activity of key cell cycle regulators via the activation of transcription factor p53 and its target genes, such as the p21 gene that codes the p21Cip1 protein [8,24]. Once activated, p21^Cip1^ triggers G_1_/S, S, and G_2_/M checkpoints, thereby blocking cell cycle progression to enable either DNA repair by specific enzymes such as PARP (poly(ADP-ribose)polymerase) proteins or cell death induction. In order to determine whether chrysin promotes cell death and senescence via the activation of the DDR pathway, we evaluated its impact on the expression of key regulators of this signaling pathway. Immunoblotting revealed that chrysin mainly induced the phosphorylation of ATM in a concentration-dependent manner and subsequently led to an increase in the downstream mediators of this pathway, such as phosphorylated forms of p53 on Ser15, which was associated with an overexpression of the p21^Cip1^ protein in the three melanic cell lines (Figure 4, Figure 5 and Figure 6). Very interestingly, we observed a decrease in ATR expression and an increase in its phosphorylated form with an overexpression of the phosphorylated form of Chk1 in SKML-28 and MelC cells (Figure 4 and Figure 5). Surprisingly, in the murine B16 cell line, the expression of the phosphorylated form of ATR was significantly decreased by the chrysin treatment, which could suggest the preponderance of the ATM pathway in these cells (Figure 6). Altogether, these results suggest that chrysin is able to induce both ATM and ATR pathways. Here, again, the appearance of the cleavage fragments of PARP demonstrates the induction of apoptosis by chrysin (Figure 4 and Figure 5).

**Figure 7:**
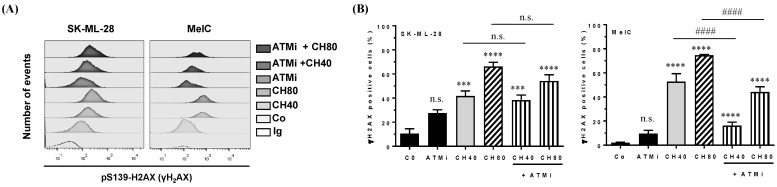
Inhibitor of the ATM pathway decreases γH2AX induction by chrysin. Melanic SK-ML-28 and MelC cells were pretreated for 6 h in the presence or absence of an ATM-specific inhibitor (ATMi, 10 µM), and then cells were treated or not with 40 (CH40) and 80 µM (CH80) of chrysin. At the end of treatments, cells were labeled with an antibody directed against γH2AX by using flow cytometry (**A**), and the percentages of positive cells were analyzed and quantified (**B**). Significant differences between treatments were determined by ANOVA, followed by Tukey’s post hoc test. n.s.: Not significant; *** *p* < 0.001; **** *p* < 0.0001 (comparison to the control); #### *p* < 0.0001.

To confirm the involvement of ATM kinase in the molecular mechanisms underlying the effects of chrysin, we treated melanoma cell lines for 72 h with polyphenol (40 and 80 µM) in the absence or presence of a specific pharmacological inhibitor of ATM (KU55933, 10 µM, ATMi). Flow cytometry histograms after the immunostaining of the γH2AX protein show the involvement of ATM in chrysin-induced DNA damage mainly in the MelC line (Figure 7A,B). Indeed, there is a slight tendency of the ATM inhibitor to decrease the percentage of γH2AX-positive cells in chrysin-treated cells at concentrations of 40 µM (Figure 7).

### 3.5. Chrysin Induces Moderate Binding to the G Quadruplex and Duplex Structures

**Figure 8:**
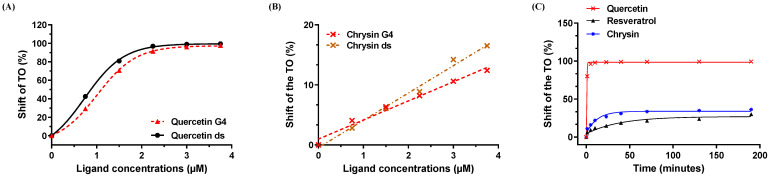
Effect of chrysin on G-quadruplexes/DNA duplexes. The ability of chrysin to bind to G-quadruplexes/DNA duplexes was assessed spectrofluorometrically using thiazole orange (TO). (**A**,**B**) DNA duplexes (ds) and G-quadruplexes (G4) (0.25 µM) were mixed with 1 µM of TO, and then increasing concentrations of quercetin (**A**) or chrysin (**B**) were added. After 3 min of equilibration, the fluorescence intensity of TO was analyzed. (**C**) G-quadruplexes (G4, 0.25 µM) were mixed with 1 µM of TO and 3.5 µM of chrysin. Changes in TO fluorescence intensity were monitored over time at regular intervals. Data are expressed as percentages of TO shift.

There is increasing evidence that the induction of severe DNA damage, DDR, and cell death may be the result of an impairment of the replication fork’s progression by particular structures, such as the G-quadruplexes (G4s) [28,29]. These G4s are non-usual DNA structures that are present in vivo in cells, folding within guanine (G)-rich sequences thanks to the innate ability of Gs to self-associate to form G-quartets, which then stack upon each other to form the columnar core of G4. The identification of ligands targeting and stabilizing these structures (G4 ligands), therefore, offers a novel anti-tumor pharmacological approach [30]. Molecules with planar aromatic structures, such as quercetin (natural flavonoid), are described as good G4 ligands [21]. We then performed in vitro affinity assays based on the displacement of a fluorescent DNA probe: thiazole orange (TO), the so-called G4-FID assay [20]. The identification of a potential ligand relies on its ability to compete with TO, resulting in a decrease in fluorescence proportional to the affinity of the compound for G4s or DNA duplexes. In these experiments, we used quercetin (3,5,7,3′,4′-pentahydroxyflavone) as a positive control because it interacts strongly with these structures. Moreover, quercetin and chrysin present an important structural homology, with both having three aromatic rings. Nevertheless, it should be noted that chrysin does not have hydroxyl groups that are similar to quercetin. In agreement with the literature, quercetin was found to be a good ligand for DNA G4s/duplexes, as evidenced by TO shift percentages (Figure 8A). Contrary to what might have been expected, chrysin showed only a moderate ability to bind to DNA, with a TO shift of less than 20% for the highest concentrations (Figure 8B). These limited effects may be explained by the absence of hydroxyl groups on the chrysin molecule. Indeed, the absence of OH^−^ groups allows the rotation of the phenolic ring, which might lead to a loss in the flatness of the molecule that is necessary for being stacked atop G4s. In another experiment, we varied the time that would allow for chrysin to adopt a better conformation, therefore resulting in better interactions. To test this hypothesis, we performed kinetics to evaluate the percentage shift of the TO of G-quadruplexes by a chrysin concentration of 3.5 µM as a function of time. Our data thus show an increase in the binding capacity of chrysin to G4 by almost 50% compared to previous experimental conditions (Figure 8C). In contrast, resveratrol, which is known to induce DDR [24], was not able to bind to G4 (Figure 8C), thus implying another mechanism of action.

### 3.6. Chrysin Decreases Key Actors of Angiogenesis Pathway

This DDR molecular pathway, via its key ATM and ATR proteins, could promote tumor angiogenesis under hypoxic conditions [31]. Chrysin has been previously shown to decrease the angiogenesis process by disrupting microvessel sprouting in human umbilical vein endothelial cells (HUVECs) or decrease neovascularization on chicken chorioallantoic membranes (CAMs) [32,33,34,35]. We thus determined the ability of chrysin to limit angiogenesis by measuring vascular endothelial growth factor (VEGF-A) secretion by using the ELISA method in both HUVECs and melanoma cells.

**Figure 9:**
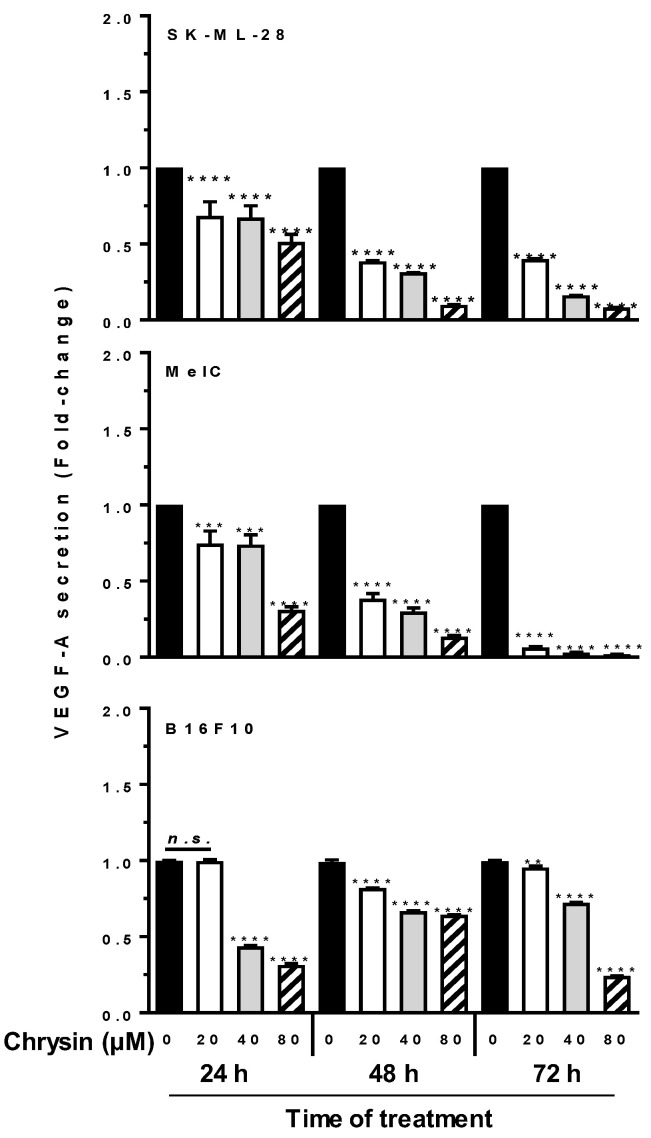
Chrysin decreases VEGF-A secretion in melanic cells. Cells were treated for 24, 48, and 72 h with a medium containing the solvent (0.1% DMSO) or with increasing concentrations of chrysin (20, 40, and 80 µM). Culture supernatants at these different treatment times were analyzed by ELISA to measure VEGF-A levels. Results are expressed as the fold change relative to control cells (DMSO). Values are the mean fold change ± SD of three independent experiments. Significant differences between treatments were determined by ANOVA, followed by Tukey’s post hoc test. ** *p* < 0.01, *** *p* < 0.001, and **** *p* < 0.0001; n.s.: not significant.

Chrysin induced a significant decrease in VEGF-A secretion in a concentration-dependent manner in most cell lines, with more marked effects after 72 h of chrysin treatment at 40 and 80 µM (Figure 9). This inhibition of VEGF-A secretion was also observed in HUVEC, a well-known in vitro model of angiogenesis, where the polyphenol reduced, in a dose-dependent manner, VEGF-A secretion that is induced by a human recombinant VEGF-165 at 50 ng/mL (Figure S1).

**Figure 10:**
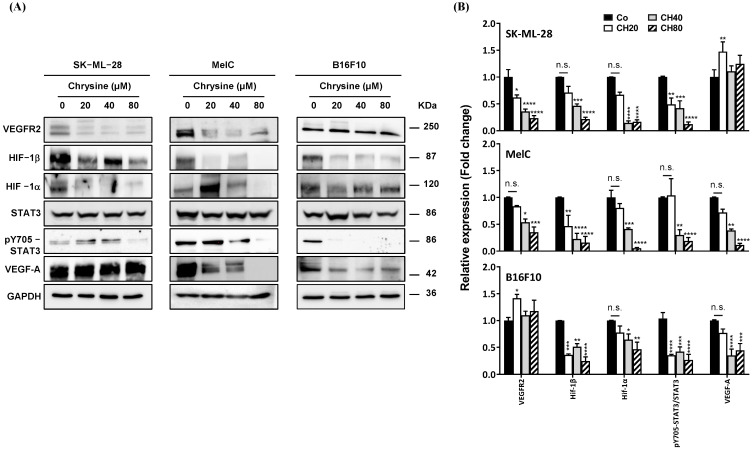
Chrysin blocks the key regulators of angiogenesis. (**A**) Immunoblot analysis of VEGF-R2, HIF-1α, HIFβ, STAT3, phospho-STAT3 (pY705-STAT3), VEGF-A in SK-ML-28, MelC, and B16F10 cells treated with a medium containing the solvent (0.1% DMSO) or with increasing concentrations (20, 40, and 80 µM) for 72 h. GAPDH was used as a loading control. (**B**) Densitometry quantification of Western blotting. Data are expressed as the mean fold induction ± SEM of three independent experiments. *p* values were determined by one-way ANOVA followed by Tukey’s multiple comparison test. * *p* < 0.05, ** *p* < 0.01, *** *p* < 0.001, and **** *p* < 0.0001; n.s.: not significant.

We then sought to determine whether this decrease in VEGF-A secretion induced by chrysin after 72 h of treatment was associated with the modulation of the expression of the key proteins involved in this process, such as the transcription factor of inducible hypoxia HIF-1α, constitutive hypoxia transcription factor HIF-1β, VEGF receptor (VEGF-R2), and transcriptional factor STAT3. As shown in Figure 10, chrysin treatment decreases the expression of VEGF-R2 in most cell lines (except for B16 cells) and hypoxia transcriptional factors (HIF1-α and HIF1β) and reduces the active phosphorylated form of STAT3 (pY705). These latter transcription factors are involved in the gene expression of VEGF-A. In agreement with the secreted levels of VEGF-A, we observed a concentration-dependent decrease in the intracellular expression of VEGF-A in MelC and B16 cell lines. However, in SKML-28 cell lines, chrysin failed to reduce the expression levels of VEGF-A (Figure 10) despite a significant decrease in its secretion (Figure 9). These results could be explained by a potential blockage of VEGF-A secretion mechanisms rather than an action on its expression level in these cells. Nevertheless, data suggest a potential antiangiogenic role of chrysin in melanoma cells.

### 3.7. Chrysin Induces ROS Production

**Figure 11:**
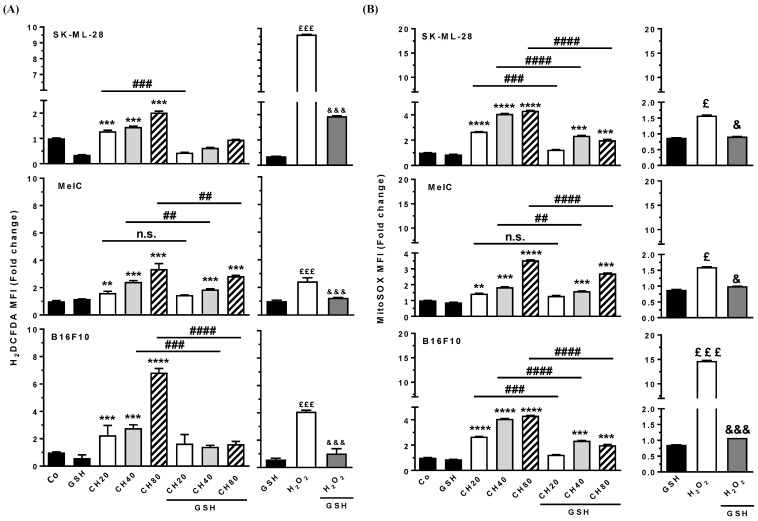
Chrysin induces oxidative stress in melanic cells. Cells were pretreated for 4 h in the absence or presence of GSH (10 mM) and then cotreated in the absence (Co, GSH) or presence of chrysin at 20 (CH20), 40 (CH40), and 80 µM (CH80) for 72 h. At the end of treatments, cells were recovered, incubated for 30 min at 37 °C in the presence of the fluorescent probes H_2_DCFA (**A**) and MitoSOX (**B**), and then analyzed by flow cytometry. The medians of fluorescence intensity (MFI) under different experimental conditions were expressed relative to the average MFI values of control cells (Co, without GSH, and without chrysin). The results are therefore expressed as a fold change relative to the control cells. Histograms represent the means of fold change ± SD, corresponding to three independent experiments (*n* = 3 per independent experiment). Probability (*p*) values were determined by an ANOVA test followed by Tukey’s post hoc test. ** *p* < 0.01, *** *p* < 0.001 and **** *p* < 0.0001 vs. control cells (Co). ##: *p* < 0.01; ###: *p* < 0.001 and ####: *p* < 0.0001 vs. chrysin treatments alone. £: *p* < 0.05; £££: *p* < 0.001 vs. GSH alone. &: *p* < 0.05; &&&: *p* < 0.001 vs. H_2_O_2_ alone. n.s.: not significant.

It is well known that oxidative stress and intracellular ROS contribute to cancer initiation and development, where in the early stages of oncogenesis, they contribute to genomic instability and malignant transformations by altering DNA repair pathways and triggering single- and double-strand breaks [36]. In order to evaluate whether chrysin-induced cell death and whether DNA damage can be associated with ROS production in melanoma cells, we analyzed the total production of ROS by flow cytometry using the fluorescent H_2_DCFDA probe (Figure 11A), and we also analyzed the production of superoxide anions by mitochondria by using the MitoSOX probe (Figure 11B). Indeed, it has been shown that cell death by apoptosis induced by polyphenols could, in part, involve the mitochondrial pathway, associated in particular with the production of superoxide anions [3,5]. Melanoma cells were therefore treated for 72 h with chrysin at 20, 40, and 80 µM alone or in the presence of an antioxidant, which comprises reduced glutathione (GSH). The cells were also incubated with hydrogen peroxide (H_2_O_2_, 40 µM, positive control) alone or in combination with the antioxidant molecule (Figure 11A,B). As observed in Figure 11A, chrysin alone induces a significant increase in global ROS production in the three cell lines compared to control cells, particularly in the B16F10 murine line at 80 µM. Similarly, there is an increase in mitochondrial superoxide anion production in response to increasing chrysin concentrations (Figure 11B). In response to H_2_O_2_, cells also produce ROS but at varying levels depending on the considered cell line (Figure 11A,B). The cotreatment of cells with chrysin and GSH confirms the ability of polyphenols to induce oxidative stress, which is partly of mitochondrial origin. Indeed, in the presence of the antioxidant, the median fluorescence intensity (MFI) of the H_2_DCFDA probe significantly decreased compared to cells treated with polyphenol alone. The same trend is observed for the MitoSOX probe (Figure 11B).

### 3.8. Chrysin Prevents Tumoral Growth and Angiogenesis In Vivo

**Figure 12:**
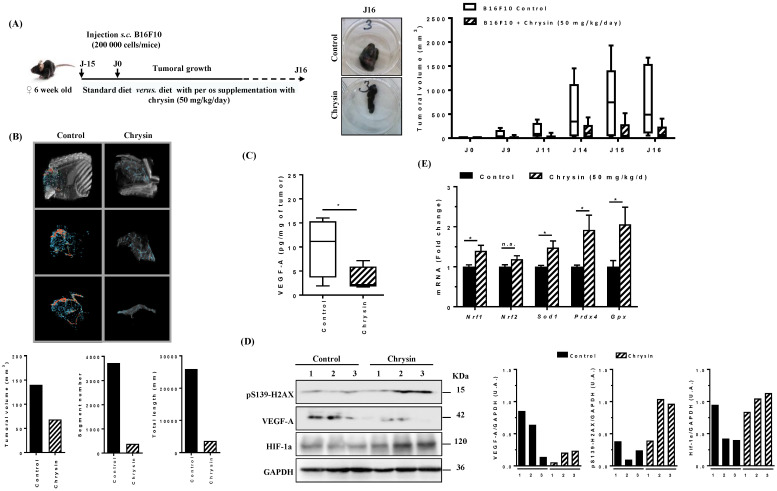
Tumor growth inhibition by chrysin in a syngeneic model of B16 melanoma. (**A**) Experimental protocol with tumor growth monitoring for 16 days after the injection of B16F10 melanoma cells (200,000 cells/mouse). (**B**) Micro X-ray CT images with a contrast agent of tumor vasculature and analyses of tumor volume, number of segments, and total length of vasculature segments. (**C**) Analysis of VEGF-A secretion by ELISA. (**D**) Immunoblotting of the DNA damage marker, i.e., 19hosphor-H2AX (pS139-H2AX), VEGF-A, and HiF-1α, in protein extracts from control and chrysin-supplemented mice. * *p* < 0.05. n.s.: not significant. Densitometry quantification of Western blotting. (**E**) Real-time qPCR analysis of angiogenesis genes (**left**) and oxidative stress genes (**right**).

In order to establish the anti-tumor potential of chrysin, we tested its efficacy in a syngeneic model of B16 melanoma in C57BL/6 mice. We measured tumor growth on 10 mice in two groups: one supplemented per os for 15 days with chrysin (50 mg/kg/day) and the other receiving a normal diet (control group) before subcutaneous injection of 200,000 B1610 cells into the mouse (Figure 12A). Tumor growth was monitored for 16 days post-tumor cell injection, and supplementation was maintained throughout the study. As observed in Figure 10A, chrysin-supplemented mice showed a slowing of growth compared to unsupplemented mice, with a significant reduction in tumor volume in chrysin-supplemented mice, as shown in the picture taken on the 16th day (Figure 12A).

To determine whether the reduction in tumors observed in the treated groups is potentially accompanied by a decrease in angiogenesis, a contrast agent was injected into the mice 14 days after tumor transplants, and the extent of vascularization was analyzed by using X-ray microscans. The results from microscanner analyses are presented in Figure 10B, and they show a decrease in both the number of vascular segments and the total length of the vascular network in chrysin-supplemented groups. The analysis of VEGF-A secretion in plasma revealed that mice supplemented with chrysin present a very low level of VEGF-A compared to untreated mice (Figure 12C). The immunoblotting of tumors evidenced that chrysin strongly decreases VEGF-A protein expression in tumors, the expression of key markers of DNA damage (pS139-H2AX), and the transcription factor of inducible hypoxia HIF-1α in tumors supplemented with chrysin compared to control mice (Figure 12D). As we have shown in vitro that DNA damage and the redox state can be linked to the antiangiogenic effect of chrysin, we highlighted that the observed in vivo effect was accompanied by a change in the expression of genes involved in the regulation of redox balance. Indeed, an RT-qPCR analysis showed that chrysin induced the expression of enzymes involved in ROS removal, such as the Nrf1 transcription factor (nuclear factor E2-related factor 1, superoxide dismutase 1 (Sod1), peroxiredoxin-4 (Prdx4), and glutathione peroxidase (Gpx) (Figure 12E).

## 4. Discussion

Melanoma is one of the cancers for which its incidence and mortality have notably increased during recent decades. According to the WHO, there are currently 2 to 3 million non-melanoma skin cancers (carcinomas) and 132,000 new malignant melanomas each year in the world. This cancer mainly affects skin cells called melanocytes, which are responsible for the secretion of melanin, a biological pigment responsible for the skin’s coloring, and it protects the DNA of skin cells from ultraviolet (UV) radiation. This form of cancer is caused and initiated mostly by excessive UV exposure, leading to the production of highly genotoxic compounds (pyrimidine cyclobutane dimers, photoproducts, and reactive oxygen species (ROS)), but it also relies on early intrinsic genetic/epigenetic changes that greatly increase the likelihood of mutations and oncogenesis [37,38]. The 10-year survival of patients with primary melanoma without metastasis with appropriate management is nearly 75–85%; however, this drops severely in patients with metastatic melanoma.

The current treatment of metastatic melanoma is based on the surgical removal of the primary tumor and its metastases if they are accessible, and radiotherapy is not applicable to this type of cancer because of well-established radioresistance. In the case of the inaccessibility and multiplicity of metastases, patients can be included in clinical trials or treated with immunotherapy, targeted therapy, or conventional chemotherapies [39]. The choice of these treatments is mainly based on the presence or absence of the mutation of the gene coding for the BRAF protein, a serine-threonine kinase involved in the activation of the MAPK (mitogen-activated protein kinase) pathway and ERK (extracellular signal-regulated kinase) 1/2 proteins, in turn transmitting signals for survival, proliferation, and invasion by the phosphorylation of various cytoplasmic and nuclear substrates. BRAF is mutated in 66% of malignant melanomas, and the mutation occurs in the kinase domain by the substitution of valine for glutamate at position 600 (V600E) in 80% of cases [40]. Thus, targeted therapies are based particularly on the use of a BRAF inhibitor (Vemurafenib) for patients with the mutation, and the targeted therapies make obtaining a significant reduction in metastases possible in 50% of cases, but resistance to treatment is observed, with relapse occurring after 7–8 months. Immunotherapy is used in stage II/III melanoma for patients without the BRAF mutation as the adjuvant treatment in addition to surgery, and it uses monoclonal antibodies directed against immune checkpoints, which comprise membrane receptors that inhibit the anti-tumor activity of T cells. These include nivolumab and pembrolizumab, which are anti-PD1 (programmed cell death protein 1) antibodies; and ipilimumab, which is an anti-CTLA4 (cytotoxic T-lymphocyte-associated protein 4) antibody [41]. The efficacy of immunotherapies is superior to that of targeted therapies; however, relapses are also observed in the medium or long term. Dacarbazine, an alkylating agent causing DNA damage, is the standard treatment recognized by ANSM (Agence Nationale de Sécurité du Médicament et des Produits de Santé) for stage IV melanoma, but it only has a low objective response rate of about 20%, with a progression-free survival of less than two months [42].

The low success rate in the treatment of melanoma is the result of the development of tumor cell resistance, particularly relative to chemotherapies. Multiple mechanisms may be at the origin of this resistance, including the following: (i) the microenvironment with respect to vascularization, diffusion, and hypoxia; (ii) pharmacokinetics with the metabolization/elimination of substances and low metabolic activation in the case of pro-drugs; (iii) the alteration of cell death pathways (e.g., apoptosis and autophagy); (iv) the alteration of tumor metabolism. The resistance of tumor cells to cell death appears to be particularly associated with alterations in DNA repair pathways, thus conferring them with the capacity for unlimited proliferation. We have previously shown that the induction and/or restoration of DNA damage can, in many cases, induce tumor cell death and sensitize them relative to therapeutic agents [8,18]. Nevertheless, under hypoxic conditions, this DDR molecular pathway, via its key ATM and ATR proteins, could promote tumor angiogenesis [31]. Conversely, the excessive production of ROS could contribute to limiting pathological angiogenesis and potentiate the effects of anti-VEGF therapies that are currently used in clinics [32]. It thus appears that oxidative stress could play a role in both the induction of DNA damage and the prevention of the development of angiogenesis, depending on the level of ROS production. The natural flavonoid, chrysin, has demonstrated that it impairs genomic stability by suppressing the repair of double-strand DNA breaks and ATM-Chk2 in various models of breast cancer cells [43,44] as well as in urinary bladder cancer cells where chrysin blocks cancer cells at the G_2_/M phase and induced global DNA hypermethylation [45]. Due to these observations, it was suggested that chrysin could act as a chemosensitizer for anticancer drugs such as doxorubicin [46], as previously observed with another polyphenol such as resveratrol in colon cancer [8]

In the present study, chrysin was used to induce an inhibition of melanic cell proliferation, for which its resistance is often associated with a loss of the DNA damage pathway. We observed that chrysin induces a blockage of tumor cells in the G_2_/M phase of the cell cycle (Figure 2) following the induction of DNA damage, as evidenced by the increase in the γH2AX marker (Figure 3). We also showed that chrysin-induced damage leads to the activation of DDR via both ATM and ATR pathways, resulting in the production of the key actors of this pathway, such as Chk1 and Chk2, and their downstream effectors, i.e., the p53 and p21 proteins (Figure 4, Figure 5 and Figure 6). This signaling pathway induced by chrysin was confirmed by the use of ATM and ATR inhibitors (Figure 7). Compared to another polyphenol such as quercetin, chrysin only showed a moderate ability to bind to DNA (both G quadruplex (G4) and duplex structures), which improves with time, suggesting that lengthening the treatment’s duration is probably required for chrysin to efficiently bind to G4s (Figure 8). This point is important since G4s are involved in many cellular processes (replication, transcription, genomic rearrangements, and telomeric dysfunctions) and prevent proper replication fork progression, which inhibits cell proliferation and induces severe DNA damage and eventually cell death. The identification of ligands targeting and stabilizing these structures (G4 ligands) offers an innovative anti-tumor pharmacological approach [20,47,48]; among them, molecules with flat aromatic structures, such as quercetin (natural flavonoid), are described as good G4 ligands [21].

**Figure 13:**
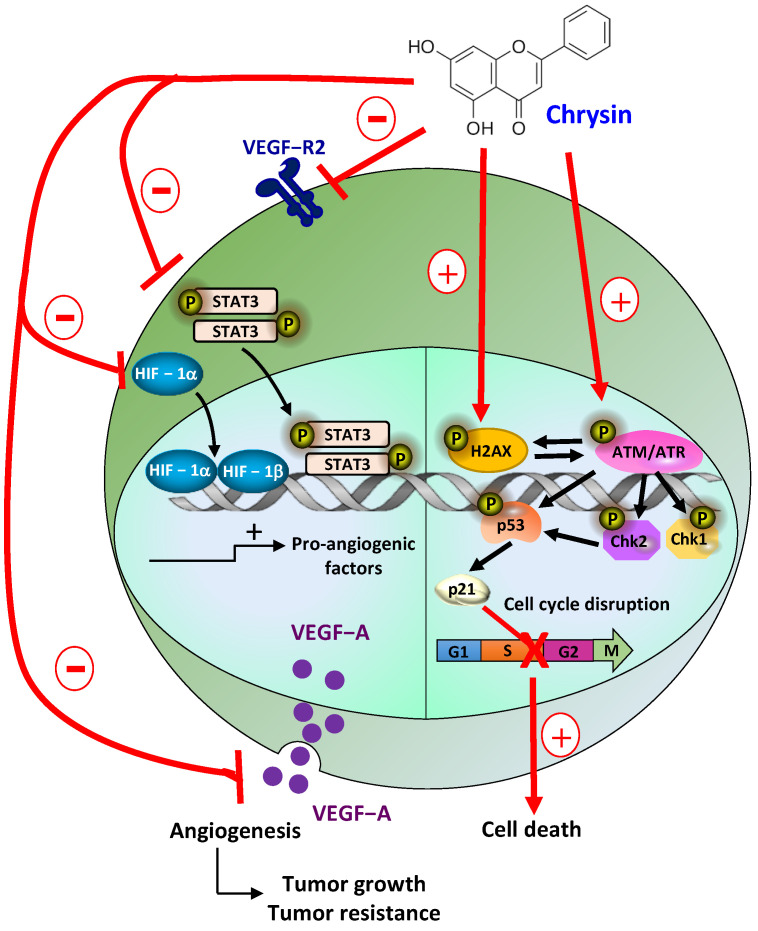
Schematic representation of chrysin action on a melanic cell. Chrysin exerts pleiotropic action on melanoma by inducing oxidative DNA damage as well as the activation of the ATM/ATR axis, which allows the activation of checkpoints Chk1 and 2, allowing the induction of the phosphorylation of p53, which can then play its role as a transcriptional factor on the P21 protein. Chrysin allows the disruption of the cell cycle in the DNA replication phase, which leads to the induction of the apoptosis of cancer cells. Furthermore, chrysin is able to reduce the process of angiogenesis and, therefore, tumor growth via its action on several factors. Indeed, chrysin first decreases the expression of VEGF-R2 but is also able to decrease the expression of transcription factors such as phosphorylated STAT3 and HIF-1. By this action on these key factors, chrysin is then able to reduce the secretion of VEGF-A in vitro and in vivo, thereby reducing the phenomenon of angiogenesis.

Here, we show that chrysin strongly induces the DDR pathway despite a moderate interaction with G4s, which leads to VEGF-A inhibition in the three tested melanoma cells (Figure 9). The decrease in these proangiogenic factors was associated with a disruption of key actors involved in its secretion, such as its VEGF-R2 receptor and the transcription factor of inducible hypoxia, HIF (Figure 10). Tumor angiogenesis is often associated with intratumoral hypoxia, which triggers the activation of HIF-1; moreover, the association of HIF-1α and HIF-1β subunits that are also inhibited by chrysin is required by the heterodimer. In a very interesting way, Okuno et al. showed the role of ATM in anti-free radical defenses and angiogenesis [49], and they suggested that molecules inducing an excessive production of ROS could contribute to limiting pathological angiogenesis and potentiate the effects of anti-VEGF therapies that are currently used in clinics. Oxidative stress thus seems to be a central component between DDR and angiogenesis, and in this context, the use of polyphenols as pro-oxidant molecules could be highly relevant. Interestingly, in the presence of GSH, the antioxidant alone reduces VEGF-A secretion, which underscores the potential role of oxidative stress in the induction of tumor angiogenesis (Figure 11). Very interestingly, the main actors in DDR, angiogenesis, and oxidative stress highlighted in the three cell lines are also affected in a syngeneic model of B16 melanoma (Figure 12). The antiangiogenic effect of chrysin was previously demonstrated mainly in two models where chrysin at 80 µM was shown to suppress both VEGF-induced angiogenesis and microvessel sprouting in human umbilical vein endothelial cells (HUVECs) [32] as well as at the basal level [33]. Moreover, other inducers of angiogenesis in this HUVEC model exhibited chrysin effects. For example, in IL-6-induced angiogenesis, chrysin was able to suppress IL-6-induced cellular migration and the formation of new vessels and branched networks in HUVECs [34]. The molecular mechanism could be a result of the downregulation of gp130/JAK1/STAT3/VEGF expression. In other models of angiogenesis, such as neovascularization on a chicken chorioallantoic membrane (CAM), chrysin significantly decreased secondary and tertiary vascular formations in a concentration-dependent manner [34] and lipopolysaccharide (LPS)-induced angiogenesis in CAM and in HUVEC [35]. In this last model, chrysin downregulates VEGF and VEGFR-2 (KDR) but not VEGFR-1 (Flt-1) gene expression. In our tumor model of melanoma, chrysin was also able to limit tumor growth by inhibiting angiogenesis and the secretion of VEGF-A and induced DNA damage (Figure 13). Furthermore, via the inhibition of VEGFR2 expression and the reduction in STAT3, chrysin could both alter the VEGF-R2 signaling pathway, involving many protein kinases such as ERK or MEK; however, this polyphenol, thanks to the suppression of phosphorylated STAT-3, could also alter the binding of the latter with the response element of the gene encoding VEGF and consequently decrease the production of VEGF, thus limiting angiogenesis.

## 5. Conclusions

Chrysin was shown to decrease angiogenesis and tumor growth in vivo in this study, which limits the progression of melanoma. Nonetheless, further investigations must be performed to demonstrate the potential use of chrysin as a nutritional complement of anti-VEGF antibodies or chemotherapy in order to counteract melanoma progression, especially in preclinical models.

## Data Availability

The authors declare that all data supporting the findings of this study are available within the article.

## Supplementary Materials

The following supporting information can be downloaded at: https://www.mdpi.com/article/10.3390/cells12121561/s1, Figure S1: Effects of Chrysin on VEGF-A secretion in Human Umbilical Vein Endothelial Cells (HUVEC); Table S1: Antibodies; Table S2: Primers used for RT-qPCR analysis.
