# Supplementary material for: Chrysin-Induced Regression of Angiogenesis via an Induction of DNA Damage Response and Oxidative Stress in In Vitro and In Vivo Models of Melanoma
Source: Cells. 2023 Jun 6;12(12):1561. doi: 10.3390/cells12121561 (PMC10297291; doi:10.3390/cells12121561)
Supplement: Supplementary file 1 [file cells-12-01561-s001.zip › cells-2361529-supplementary.pdf]

Supplementary materials

Supplementary Table S1. Antibodies

Epitope	Supplier	Reference	Isotype	kDa	Dilution	Dilutent
ATM	Cell signaling	#2873	Rabbit	350	1/1000	BSA 5%
pS1981-ATM	abcam	Ab 81292	Rabbit	370	1/50 000	BSA 5%
ATR	Santa Cruz	Sc-1887	Chèvre	250	1/200	Milk 5%
pS428-ATR	Cell signaling	#2853	Rabbit	300	1/1000	BSA 5%
Chk1	Santa Cruz	Sc-8408	Mouse	56	1/1000	Milk 5%
pS345-Chk1	Cell signaling	#2341	Rabbit	56	1/1000	BSA 5%
Chk2	Millipore	05-649	Mouse	67	1/1000	Milk 5%
pT68-Chk2	Abcam	Ab 3501	Rabbit	61	1/500	Milk 5%
p53	Cell signaling	#2527	Rabbit	53	1/1000	BSA 5%
pS15-p53	Cell signaling	#9284	Rabbit	53	1/1000	Milk 5%
$p21^{Cip1}$	Santa cruz	Sc-756	Rabbit	21	1/500	Milk/BSA 2,5%
pS139-H2AX (γH2AX)	Santa cruz	Sc-10169	Rabbit	15	1/500	BSA 5%
PARP	Cell signaling	#9542	Rabbit	116,89	1/1000	Milk 5%
GAPDH	Sigma Aldrich	G9545	Rabbit	36	1/1000	Milk 5%
VEGFR2	Cell signaling	#2479	Rabbit	210,230	1/1000	BSA 5%
Hif1α	Cell signaling	#14179	Rabbit	120	1/1000	BSA 5%
Hif1β	Abcam	Ab 2771	Mouse	87	1/4000	Milk 5%
STAT3	Cell signaling	#4904	Rabbit	79,86	1/2000	BSA 5%
pY705-STAT3	Cell signaling	#9145	Rabbit	79,86	1/2000	BSA 5%
VEGF-A	Santa cruz	Sc-7269	Mouse	21,42	1/200	Milk 5%
Anti-Mouse IgG HRP	Sigma Aldrich	A0168	Goat		1/10000	Milk 5%
Anti-Rabbit IgG HRP	Cell signaling	#7074	Goat		1/2500	Milk 5%
Anti-Goat IgG HRP	Dako	PO449	Rabbit		1/4000	Milk 5%

Supplementary Table S2. Primers used for RT-qPCR analysis

Gene	Species	Forward sequence	Reverse sequence
***VEGFA* (VEGF-A)**	Human	TTCATGGATGTCTATCAGCG	CATCTCTCCTATGTGCTGGC
***GAPDH* (GAPDH)**	Human	AGATCCCTCCAAAATCAAGTGC	GGCAGAGATGATGACCCTTTT
***Vefgr1* (VEGFR2)**	Mouse	GAGGAGGATGAGGGTGTCTAT	GTGATCAGCTCCAGGTTTGAC
***Vefgr2* (VEGFR2)**	Mouse	GCAAAACACTCACCATTCCCA	GAGGTTTGAAATCGACCCTCG
***Vegfa* (VEGF-A)**	Mouse	AATGCTTTCTCTGAA	GCTTCCTACAGCACAGCACA
***Hif1a* (HIF1α)**	Mouse	TCTCGGCGAAGCAAAGAGTC	AGCCATCTAGGGCTTTCAGATAA
***Epas1* (HIF2α)**	Mouse	ACTGGGAGGCATAGCACTGT	TAAAGCGGCAGCTGGAGTAT
***Stat3* (STAT3)**	Mouse	CTGCTCCAGGTAGCGTGTGT	CTCAGCCCCCGGAGACAGT
***Nrf2* (NRF2)**	Mouse	TAGATGACCATGAGTCGCTTGC	GCCAAACTTGCTCCATGTCC
***Nrf1* (NRF1)**	Mouse	AGCACCGAGAGACCCAAAC	TGTACGTGGCTACATGGACCT
***Prdx4* (PRDX4)**	Mouse	CCACCAGCGTAGAAGTGGC	GTTCCTGTTGCGGACCGAAT
***Cat* (CATALASE)**	Mouse	GCTGAAGTTGAACAGATG	GTCATCAGCGTGAGTCTG
***Gpx* (GPX)**	Mouse	CCCTAGGAGAATGGCAAG	CAGAGTGCAGCCAGTAATCACCAAG
***Sod1* (SOD1)**	Mouse	GACCTGGGCAATGTGACTGCTG	CACCAGTGTACGGCCAATGATG
***Gapdh* (GAPDH)**	Mouse	GTTGTCTCCTGCGACTTCA	GGTGGTCCAGGGTTTCTTA

Supplementary Figure S1.

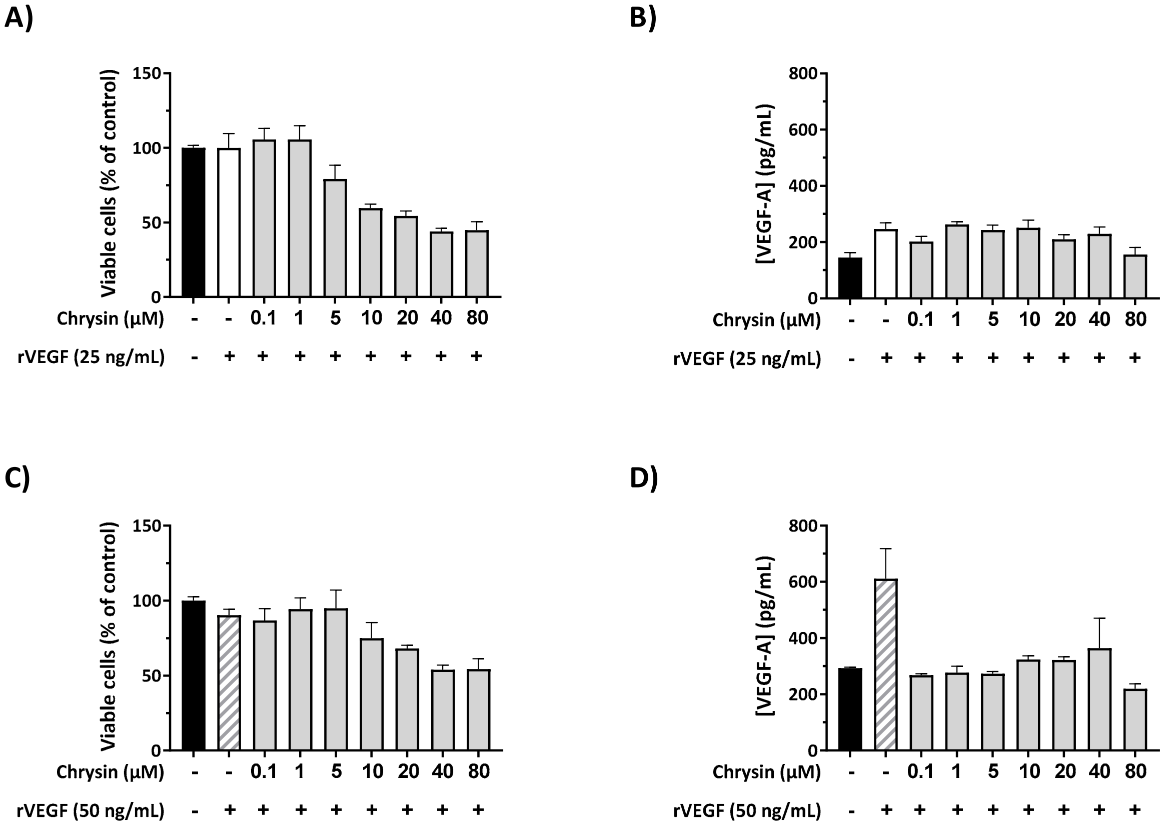

Supplementary Figure S1. Effects of Chrysin on VEGF-A secretion in Human Umbilical Vein Endothelial Cells (HUVEC). HUVEC cells were seeded in 48-well plates (15,000 cells per well) in EBM media (CC-3129 without phenol red, Lonza®) supplemented with EGM SingleQuots (CC-4133, Lonza®) and incubated 24h for cells adhesion. The next day, cells were pretreated without (vehicle control, DMSO 0.05% v/v) or with increasing concentrations of chrysin (0.1, 1, 5, 10, 20, 40 and 80 µM) for 24h. After chrysin pretreatment, cells were either incubated for additional 24h with human recombinant VEGF-165 (rVEGF, 11858821, Invitrogen) at 25 (**A, B**) or 50 ng/mL (**C, D**), or treated with vehicle alone (0.1 % water). At the end of the experiment, cells supernatants were collected and the secreted levels of VEGF were quantified by ELISA (Human VEGF ELISA MAX™ Deluxe set, 446504, Biolegend). **B and D**), Data are expressed as a mean ± SEM in pg/mL from one independent experiment with 3 biological replicates per condition. **A and B**), cells viability was assessed in treated wells by crystal violet staining. Data are expressed as a mean percentage relative to control cells (treated with vehicles alone) ± SEM.
